# The Role of Endoscopy in the Postoperative Management of Patients Treated for Esophageal Atresia: 20 Years of Experience

**DOI:** 10.3390/diagnostics15070843

**Published:** 2025-03-25

**Authors:** Francesco Grasso, Fabio Baldanza, Sara Pernicone, Marco Pensabene, Maria Sergio, Maria Rita Di Pace

**Affiliations:** Pediatric Surgical Unit, Department Health Promotion of Mother and Child Care Internal Medicine and Medical Specialties, University of Palermo, 90127 Palermo, Italy; fabio.baldanza@policlinico.pa.it (F.B.); sarapernicone@gmail.com (S.P.); marco.pensabene@policlinico.pa.it (M.P.); sergio.maria@policlinico.pa.it (M.S.)

**Keywords:** endoscopy, esophageal atresia, esophageal stricture, esophagitis, gastroesophageal reflux

## Abstract

**Background/Objectives**: Endoscopy plays a well know role in managing patients treated for esophageal atresia (EA), allowing the detection and treatment of complications such as anastomotic strictures, gastroesophageal reflux disease, and other structural abnormalities, during the critical first year of life. Nevertheless, we would like to underline the importance of endoscopy early in the follow-up of patients treated for EA, as recommended by guidelines. This study evaluates the role of endoscopy in managing patients treated for esophageal atresia (EA), focusing on the detection and treatment of complications such as anastomotic strictures, gastroesophageal reflux disease, and other structural abnormalities during the critical first year of life. **Methods**: A retrospective analysis was conducted over 20 years at our institution. Clinical assessments were performed at 3, 6, and 9 months to monitor growth, feeding tolerance, and proton pump inhibitor (PPI) adjustments. Endoscopic evaluations were conducted under general anesthesia around one year of age. **Results**: Between 2003 and 2023, 84 patients underwent surgical treatment for EA, with complete follow-up data available for 77 patients. Complications occurred in 21 patients (27%), including 4 patients (5.5%) with isolated anastomotic stricture, 8 patients (10%) with reflux esophagitis, 8 patients (10%) affected by both stenosis and reflux, and 1 case (1.5%) of fistula recurrence. Endoscopic dilatations for stenosis were successful, averaging three procedures per patient. Growth parameters were normal in 91% of cases by the first year. **Conclusions**: Esophagogastroscopy is a safe and effective tool for diagnosing and managing complications after EA repair in infants. The minimally invasive procedure could allow early detection of esophagitis and strictures, offering significant therapeutic benefits. Given these important results, we would like to recommend its use in routine follow-up care.

## 1. Introduction

Esophageal atresia (EA) is a congenital condition characterized by an incomplete formation of the esophagus, often associated with tracheoesophageal fistula (TEF). Surgical intervention is typically required to correct these anomalies. Although many patients achieve favorable outcomes, the postoperative period can be complicated by various issues, including anastomotic strictures, gastroesophageal reflux disease (GERD), and other structural abnormalities, that can significantly impact the long-term health and quality of life of these patients [1].

The first year of life is particularly vulnerable since it is fundamental for growth, development, and the establishment of feeding pathways, including patients treated for EA. Furthermore, at this age some symptoms may often be misrecognized or overlooked, and the early detection and management of such different conditions improve the outcome. Endoscopy has emerged as a well-tolerated tool in the diagnostic and therapeutic management of patients after EA correction [2].

This study aims to evaluate the role of endoscopy in the postoperative management of patients treated for EA over a 20-year period at our institution. By examining the frequency of complications and the success of endoscopic procedures, we aim to offer valuable perspectives on the role of this technique in improving patient management and achieving better long-term outcomes for individuals with EA.

## 2. Materials and Methods

The study aims to correlate gastro-esophageal endoscopy and clinical signs and symptoms in patients treated for EA. We enrolled patients who completed clinical and instrumental follow-up up to 1 year of age over the past 20 years at the Pediatric Surgery Unit of “Paolo Giaccone” University Hospital in Palermo. Preoperative evaluation consisted of different parameters such as a prenatal diagnosis, gestational age at delivery, birth weight, prognostic risk classification according to Spitz, and suspected type of esophageal atresia according to Gross. The intraoperative variables considered were bronchoscopy, the type of EA, and the subsequent surgical approach. Bronchoscopy allowed us to assess the type of esophageal atresia and associated airway anomalies. After esophageal repair, all patients received PPI therapy within the first year of life. All patients underwent clinical monitoring in an outpatient setting at 3, 6, and 9 months. The clinical follow-up aimed to assess growth, respiratory, and gastrointestinal symptoms, such as cough, vomiting, and poor feeding tolerance. Simultaneously, PPI medication was modulated according to weight and gradually reduced and suspended one month before the planned endoscopy; it was resumed in case of symptoms and/or esophagitis. Endoscopic examination with biopsies was performed using a flexible gastroscope under general anesthesia within the first year of life. The time of endoscopy was anticipated in symptomatic patients to allow appropriate diagnostic and therapeutic management.

Endoscopy was performed to study the macroscopic appearance of the upper esophagus, the patency of the lumen, the esophago-gastric junction, the anastomotic site, and the stomach aspect. Moreover, the procedure gave us indirect information on the functional behavior of the lower esophageal sphincter (LES) based on the ability of the gastroscope to pass the LES and for the LES itself to properly close around it.

A minimum of four biopsies were performed in the distal esophagus for histological characterization.

When an esophageal stricture was detected, a gastric guidewire was concurrently positioned through the stenotic tract, and the passage of the semirigid Savary-Gilliard dilators into the proximal esophageal lumen was monitoring under endoscopic vision.

During endoscopy, bronchoscopy was performed in patients with persistent respiratory symptoms. According to endoscopic and pathologic results, clinical symptoms were related to the occurrence of esophageal strictures, esophagitis, and anomalies of feeding pathways. Chi-square and *T* tests were used to perform statistical analysis using a free software available online, and significance was considered with a *p*-value lower than 0.05.

## 3. Results

From 2003 to 2023, 84 patients underwent surgical treatment for EA at our institution. Seven of them (9%) died of major malformations, in the absence of reported surgical complications, at least one month after surgical repair. Of 77 patients with a complete clinical and instrumental follow-up, 43 were males (56%), and 34 were females (44%). In 47 patients (61%), prenatal ultrasound showed signs suggesting esophageal atresia, including polyhydramnios and the absence of a gastric bubble. A total of 28 patients (36%) were born preterm, with a mean gestational age of 34 weeks (range 28–36), and 49 patients (64%) were born at term. The average birth weight was 2665 g (range 700–4150 g).

Preoperative bronchoscopy showed an isolated lower fistula in 64 (83%) cases. Six (8%) patients presented with double fistulas, while no fistula was detected in the remaining seven (9%) patients.

Patients were consequently classified according to the Gross system, resulting in a total of 64 (83%) Type C; 7 (9%) Type A, including 2 long-gap cases; and 6 (8%) Type D. Using Spitz’s prognostic classification, we found 66 patients (86%) in Class I, 8 patients (10%) in Class II, and 3 patients (4%) in Class III (Table 1).

Associated defects were found in 22 patients (29%), as summarized in Table 2.

All patients underwent thoracotomy for end-to-end esophago-esophageal anastomosis with or without closure of the tracheoesophageal fistula. The two patients with long-gap esophageal atresia underwent cervical esophagostomy and gastrostomy, followed by esophageal replacement surgery within the first six months of life, including gastric pull-up in one case and colonic transposition in the other one.

All patients received therapy with proton pump inhibitors until 1 month before the endoscopic procedure. No patient reported anesthetic complications during endoscopic procedures under general anesthesia. A total of 21 patients (27%) experienced complications. Of these, 8 patients (10%) had isolated reflux esophagitis, 8 patients (10%) presented with both stenosis and reflux, 4 patients (5.5%) with isolated anastomotic stricture, and 1 patient (1.5%) developed fistula recurrence (Table 3).

None of these patients experienced major symptoms requiring hospitalization. Furthermore, endoscopy was anticipated at about 6–10 months of life in 12 (15%) patients with persistent minor symptoms such as cough, wheezing, vomiting, frequent regurgitation, and poor feeding tolerance.

Growth curves were within normal percentiles by the first year of life in 70 patients (91%). In the remaining seven (9%) patients, the growth curve was reduced below the 25th percentile. Three of these patients (4%) had an anastomotic stricture, and four (5%) presented other pathologic conditions or syndrome (Table 1).

All symptomatic patients presented with anastomotic stricture, including 8 patients with associated reflux, and the remaining four with the condition as an isolated condition. A significant relation was found between stenosis and symptoms occurrence (*p* < 0.0001); nonetheless, the association between isolated esophagitis and related symptoms was not significant (*p* = 0.44) (Table 4).

All cases of stenosis were successfully treated with endoscopic dilations in general anesthesia with semirigid Savary-Gilliard dilators. The average number of endoscopic dilations needed to resolve esophageal stricture was three (range 1–5), performed every 2–3 weeks. Isolated episodes of mild bleeding were recorded that resolved spontaneously. Fistula recurrence was managed with reoperation.

**Figure 1:**
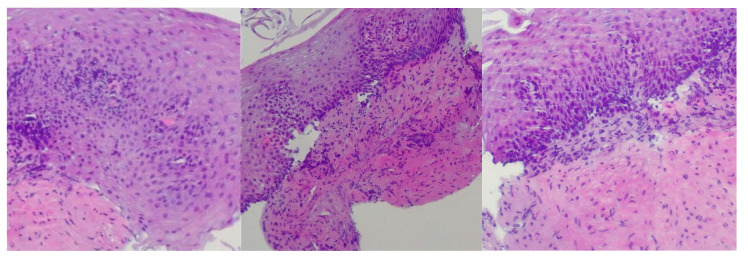
Microscopic signs of reflux esophagitis. Biopsy findings showing stratified squamous epithelium, basal layer hyperplasia, chronic inflammatory infiltrate in the lamina propria, acanthosis, papillomatosis, and focal neutrophilic granulocytic infiltration within the papillary axes. The images, from left to right, are magnified at ×400, ×200 and ×400.

Reflux esophagitis was confirmed by biopsy findings showing stratified squamous epithelium, basal layer hyperplasia, chronic inflammatory infiltrate in the lamina propria, acanthosis, papillomatosis, and focal neutrophilic granulocytic infiltration within the papillary axes (Figure 1).

Patients with reflux esophagitis continued PPI therapy until the subsequent endoscopic follow-up.

No patient presented with eosinophilic esophagitis.

The overall mean age at weaning was 6.6 months of age (range 4–12), but a significant difference was observed in patients with and without stenosis, undergoing weaning at 8.5 (range 6–10) and 6.35 (range 4–12) months (*p* < 0.0003), respectively (Table 4).

A total of 19 patients (25%) were lost to clinical and instrumental follow-up after the first year.

## 4. Discussion

Esophageal atresia (EA) is a congenital malformation characterized by a discontinuity of the esophagus, often associated with tracheoesophageal fistula (TEF). The incidence of EA is 1 in every 2500–4000 live births [3].

Surgical operation aims to restore the structural integrity of the esophagus and close the fistula when present. Since the 1940s, when the fist repair was performed, significant advancements have been made in surgical repair techniques and the multidisciplinary management of neonates with EA. Today, the overall survival rate is approximately 95%, although it seems to be reduced to 90% in cases of severe associated malformations and/or extremely low birth weight [4].

The exact etiology still remains unknown, despite some theories having been postulated. Other anomalies are detectable in over 50% of patients, which may include cardiac, vertebral, anorectal, renal, and limb defects. It can be observed as a manifestation of VACTERL association or other rare syndromes. Despite the good prognosis, morbidities following surgical repair remain challenging. These predominantly include respiratory issues, gastroesophageal problems, and growth disturbances; complications like esophagitis, gastroesophageal reflux disease (GERD), recurrent TEF, esophageal stenosis, and respiratory infections are frequently observed in EA patients [5,6,7].

Esophagogastroscopy has been safely performed under general anesthesia in infants with esophageal atresia (EA) within the first year of life to promptly identify any issues, enabling a personalized therapeutic approach. Over the past three decades, gastrointestinal endoscopy in the pediatric population has significantly advanced, leading to an increase in both diagnostic and therapeutic applications. Enhancements in endoscope design and the development of specialized endoscopic devices have played a crucial role in this evolution. Thanks to advancements in gastrointestinal endoscopy and anesthesia, even premature infants and critically ill patients can undergo examinations starting from their first day of life [8].

**Figure 2:**
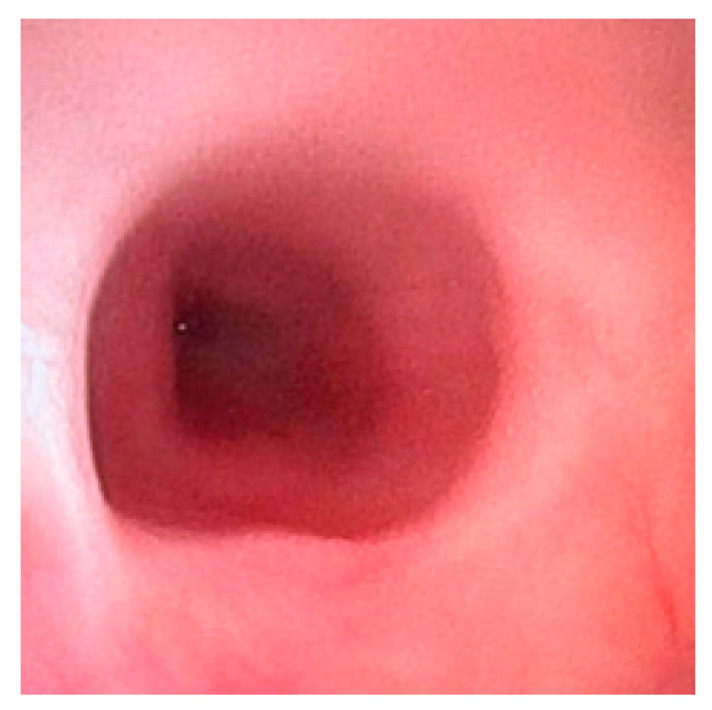
Site of the previous esophageal anastomosis with mild caliber reduction and without evidence of stenosis. Good distensibility on insufflation and smooth instrument passage. Mild caliber discrepancy between the upper and lower stumps.

**Figure 3:**
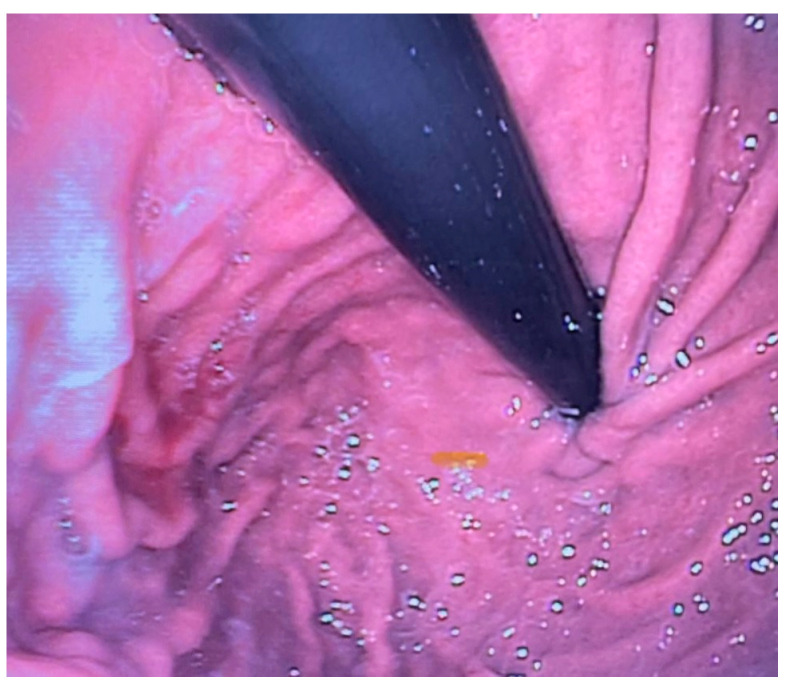
Normal LES contractility on endoscopic examination.

Esophagogastroscopy allowed us to obtain multiple pieces of information in our patients: it allowed us to assess both esophageal and gastric anatomy under direct vision, as well as the contractility of the lower esophageal sphincter. It also provided insights into the macroscopic characteristics of the esophageal and gastric mucosa (Figure 2 and Figure 3). Moreover, by performing at least four esophageal biopsies during each procedure, we were able to confirm the presence of esophagitis and classify its type, which could be a potential consequence of gastroesophageal reflux disease (GERD), using histopathological analysis [9].

**Figure 4:**
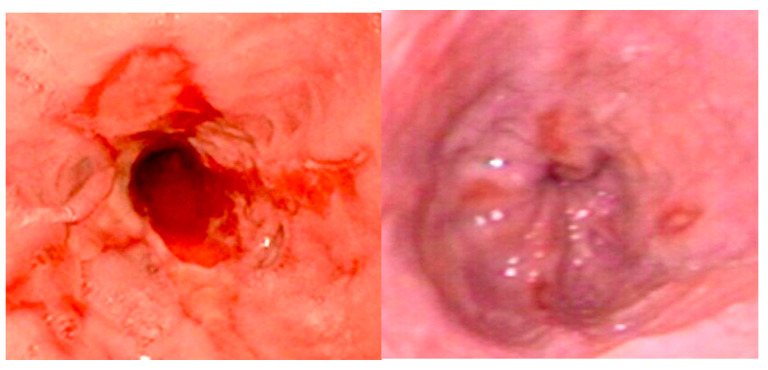
Macroscopic signs of reflux esophagitis.

Gastroesophageal reflux (GER) is defined as the backflow of stomach contents into the esophagus, with or without regurgitation or vomiting. It is considered pathological when it leads to bothersome symptoms or complications such as stenosis, pulmonary consequences, or esophagitis (Figure 4) [10].

In clinical practice, and particularly when assessing infants, it can be challenging to distinguish gastroesophageal reflux (GER) from gastroesophageal reflux disease (GERD). This is especially true for nonverbal infants, where identifying troublesome symptoms could be more complex. The manifestations of infant GERD can vary and be unspecific, including excessive crying, back arching, poor weight gain, regurgitation, and irritability. However, many of these signs are also common in healthy infants, with or without GERD, which can make a proper diagnosis difficult. Furthermore, the terms GER and GERD are often used interchangeably by both healthcare providers and parents, further complicating the clinical picture [11].

Consequently, differentiating between GER and GERD in children is often challenging, making it difficult to accurately identify infants and young patients affected by GERD and to determine its actual prevalence and clinical significance [7].

The prevalence of GERD in patients treated for EA is significantly higher than in the general population, ranging from 26% to 70% in different studies, depending on the type of reflux evaluation and the patient’s age. GER is thought to be a consequence of the initial surgery, especially during the creation of the anastomosis, because the mobilization of the esophageal pouches may impair esophageal motility and compromise the natural anti-reflux mechanisms [5,12].

Due to the high prevalence and associated complications of GERD, the ESPGHAN/NASPGHAN consensus panel advises that proton pump inhibitors (PPIs) should be administered in these patients until they reach one year of age, with continued monitoring for acid reflux in older patients. Nonetheless, the clinical advantages of PPI prophylaxis remain ambiguous, and the long-term use of these medications should be evaluated with caution, weighing the potential benefits against the risks of adverse effects [13].

In patients older than one year of age, the occurrence of esophageal atresia linked to significant gastroesophageal reflux increased only slightly, and only a small number of additional patients required anti-reflux surgery. These patients should be evaluated carefully, as it may worsen dysphagia or esophageal motility issues [14].

The current literature indicates that the prevalence of gastroesophageal reflux disease (GERD) and eosinophilic esophagitis (EoE) increases with age; however, we did not identify EoE in the presented series.

Isolated reflux esophagitis was observed in eight asymptomatic patients, suggesting that isolated esophagitis not associated with stenosis may go unnoticed or subclinical. This finding reinforces the importance of endoscopy as a screening tool to identify asymptomatic patients with esophagitis who may benefit from PPI therapy.

Currently, there is no established gold standard diagnostic tool for gastroesophageal reflux disease (GERD) in infants and children. In the presented series, patients with reflux esophagitis continued PPI therapy until the subsequent endoscopic follow-up. The regimen of anti-reflux therapy with proton pump inhibitors (PPIs) and additional medications has been developed and modified according to personalized clinical and instrumental findings, specifically tailored to the patient [13].

**Figure 5:**
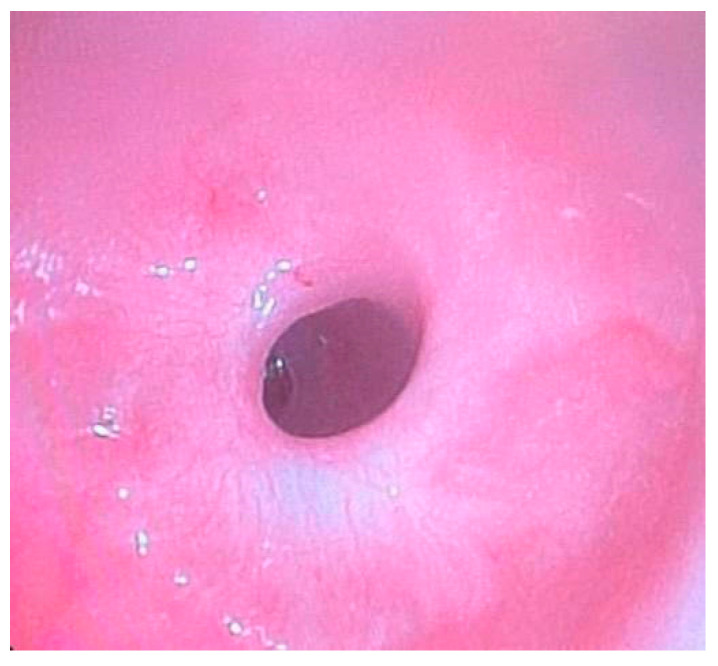
Esophageal anastomotic stricture in an AE patient.

Anastomotic stricture, a circumferential narrowing of the esophageal lumen, occurs in up to 50% of patients treated for EA, and may significantly impact respiratory function and feeding processes from the early postoperative period. The main risk factors involved in anastomotic stricture occurrence are gastroesophageal reflux (GER), tension at the anastomosis site, and the presence of long-gap atresia (Figure 5) [15].

These strictures can lead to a range of symptoms, including dysphagia, vomiting, and feeding difficulties, which are critical to monitor during follow-up. Interestingly, some patients, especially neonates and infants dependent on formula or breast milk, may present with atypical symptoms or none at all, which underscores the necessity for vigilant observation of their overall health [14,15,16].

A variable incidence of esophageal strictures between 18% and 50% has been reported, with patients experiencing the onset of this complication within the first year following surgical intervention. When treatment is required, endoscopic esophageal dilation remains the primary approach [17].

In our study, anastomotic stricture was identified in 12 patients (15.5%), all of whom experienced mild gastrointestinal or respiratory symptoms, highlighting a strong association between stricture formation and symptom occurrence (*p* < 0.0001). Additionally, reflux esophagitis was diagnosed in eight of these patients, further supporting the well-established link between these two conditions.

Esophageal strictures are among the most prevalent conditions necessitating endoscopic intervention in pediatric patients. Both balloon and semirigid dilators are frequently employed for the dilation of esophageal strictures also in children, with no significant differences in terms of efficacy or complications. Balloon dilators predominantly utilize radial force, thereby avoiding the simultaneous application of axial force, which occurs with semirigid dilators. The experience of the operator is essential to ensure that the insertion of the dilator is performed without excessive force or haste [18].

**Figure 6:**
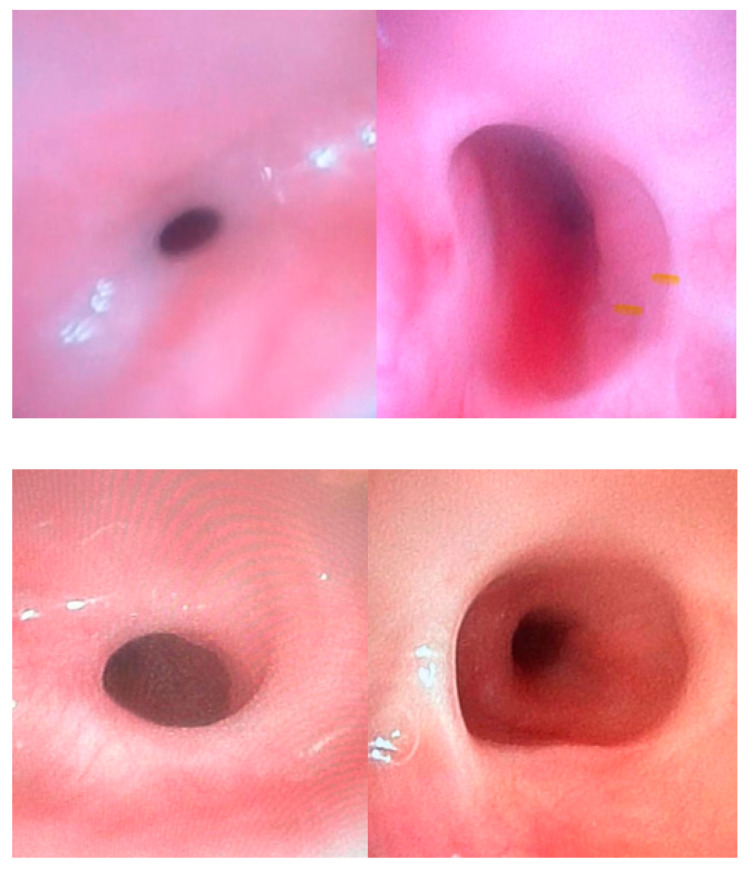
Esophageal anastomotic strictures before and after endoscopic dilation program.

Patients with esophageal stenosis may require multiple dilation procedures due to the recurrent nature of these strictures. Most studies have used a minimum interval of 3 weeks between dilation sessions, with an average of three dilations typically required (Figure 6) [19].

Endoscopic steroid injection, systemic steroid therapy, and the topical application of mitomycin C are emerging strategies aimed at improving the effectiveness of dilation and enhancing the management of recurrent and refractory esophageal strictures [20].

Nevertheless, diagnostic esophagogastroscopy allowed us to identify esophagitis and esophageal strictures and simultaneously provide treatment, saving the patient from ionizing radiation. It remains one of the most commonly performed endoscopic procedures in the pediatric population. Complications such as bleeding and perforation are rare, while transient symptoms, such as sore throat or hoarseness, are commonly observed following the procedure in children and usually resolve spontaneously without specific treatment.

Among the most severe complications of esophageal dilation is esophageal perforation. The reported incidence of perforation following esophageal dilation in congenital esophageal atresia is higher (10–44%) than that observed for other types of esophageal stenosis in children, likely due to the increased resistance of the stenosis and the risk of sudden rupture during dilation [15].

In our experience, no cases of esophageal perforation occurred during endoscopic dilations, and all strictures were successfully treated endoscopically with a mean of three dilatations (range 1–5), which is consistent with literature. This suggests that a structured follow-up, which involves proton pump inhibitor (PPI) therapy and routine endoscopic evaluations within the first year of life, played a key role in the early detection of esophageal stenosis. By enabling timely intervention, this comprehensive strategy enhances both the safety and efficacy of treatment, reducing the need for alternative therapeutic measures such as esophageal stent placement or percutaneous endoscopic gastrostomy (PEG) for nutritional support and ultimately improving patient outcomes [21].

Recurrent tracheoesophageal fistula (TEF) is a rare but severe complication that necessitates prompt surgical intervention to prevent serious respiratory complications. The incidence reported in the literature ranges from 1.9% to 11%. Although some complications may present early in life, recurrent TEF can manifest later, underscoring the importance of ongoing surveillance. Early detection is crucial to mitigate the risk of significant morbidity. Esophagogastroduodenoscopy, preferably in combination with bronchoscopy, can be considered a valuable diagnostic tool in cases of TEF. Closure of the fistula may be performed endoscopically; however, this approach often requires multiple attempts and carries inherent risks. The optimal technique for repairing recurrent fistula remains a matter of debate among clinicians, highlighting the need for individualized treatment strategies to ensure the best outcomes for patients [4,22,23].

## 5. Conclusions

Since complications in patients with EA may be asymptomatic, particularly in nonverbal infants, a multidisciplinary surveillance approach combined with endoscopic procedures is crucial for improving management and long-term outcomes. This approach addresses the complex interplay among surgical, gastrointestinal, nutritional, and respiratory issues that impact recovery and development. Regular endoscopic follow-up, as recommended by NASPHGHAN-ESPGHAN guidelines, is essential for the early detection and management of complications. Endoscopy in the comprehensive care of pediatric patients with esophageal atresia may be preferred as a diagnostic or therapeutic option in place of procedures that require ionizing radiation. Furthermore, this procedure allows for the assessment of disease severity and progression.

Esophagogastroscopy, when performed by skilled endoscopists, is a safe and efficient method for diagnosing and treating complications in pediatric patients post-EA surgery.

Nevertheless, endoscopy is an invasive procedure that requires anesthesia in pediatric patients, which is generally safe but carries inherent risks that must be carefully assessed. Given the size of our sample, additional studies are necessary to further highlight the importance of endoscopy in the follow-up care of patients treated for esophageal atresia.

## Figures and Tables

**Table 1:** Descriptive statistic findings.

Preoperative	**(n)**	
	Sex	Males 43 (56%)
		Females 34 (44%)
	Gestational age	Preterm 28 (36%)
		Term 49 (64%)
	Prenatal diagnosis	Yes 47 (61%)
		No 30 (39%)
	Gross system	Type A 7 (9%)
		Type C 64 (83%)
		Type D 6 (8%)
Postoperative	**(n)**	
	Growth curves	Normal 70 (91%)
		Abnormal 7 (9%)
	Follow-up after first year	Follow 58 (75%)
		Lost 19 (25%)
	Persistent minor symptoms	Yes 12 (15.5%)
		No 65 (84.5%)
	Endoscopy dilatation	Needed 12 (15.5%)
		Unneeded 65 (84.5%)

**Table 2:** Associated congenital defects and syndromes.

	Malformations/Syndromes	Case (n)	%
Gastrointestinal	Anorectal malformations	10	41
	duodenal atresia	2	
Respiratory	Bilateral vocal cord hypomobility	1	7
	Choanal atresia	1	
Cardiological	Atrial septal defect (ASD)	2	17
	Patent foramen ovale (PFO)	2	
	Right-sided aortic arch	1	
Musculoskeletal	Polydactyly	1	14
	Vertebral structural anomalies	1	
	Sacral agenesis	1	
	Supernumerary ribs	1	
Urinary	Bilateral vescicoureteral reflux (VUR)	1	4
Chromosomal disorder	Cystic fibrosis	1	17
	Fanconi anemia	1	
	Alport syndrome	1	
	Nager syndrome	1	
	Goldenhar syndrome	1	

**Table 3:** Postoperative complications.

Complications	Patients (n)	%
MRGE	8	10
Esophageal stricture	4	5.5
MRGE + esophageal stricture	8	10
Fistula recurrence	1	1.5

**Table 4:** Correlation between symptoms and the presence of esophageal stenosis and/or esophagitis.

Group	With Stricture	Without Stricture	*p*-Value
Mean age at weaning (months)	8.5 (range 6–10)	6.35 (range 4–12)	<0.0003
Symptoms (n)	12/12 (100%)	0/65 (0%)	<0.0001
Esophagitis (n)	8/12 (66%)	4/12	0.44

## Data Availability

The data presented in this study are available from the corresponding author upon request.
